# A Very Rare Case: HPV-Negative Vulvar Cancer in an Adolescent

**DOI:** 10.1155/2018/1816782

**Published:** 2018-02-01

**Authors:** Ilker Kahramanoglu, Hasan Turan, Yahya Ozgun Oner, Tugan Bese, Sennur Ilvan, Macit Arvas, Fuat Demirkiran

**Affiliations:** ^1^Cerrahpasa Faculty of Medicine, Department of Obstetrics and Gynecology, Division of Gynecologic Oncology, Istanbul University, Istanbul, Turkey; ^2^Cerrahpasa Faculty of Medicine, Department of Pathology, Istanbul University, Istanbul, Turkey

## Abstract

Carcinoma of the vulva is usually regarded as a disease of older women, with the typical age of 65–85 years. There are a limited number of reports of vulvar cancer cases younger than 30 years. These patients have usually risk factors such as human papillomavirus (HPV) infection and immunosuppression. Herein, we present a case of invasive squamous vulvar cancer in an 18-year-old patient without any risk factor. Vulvar radical local excision and bilateral inguinal sentinel lymph node biopsies were performed. The clitoris was preserved during the surgery. Patient did not receive adjuvant therapy. Follow-up after 12 months of the disease showed no evidence of disease. Vulvar carcinoma in very young women may develop without any predisposing factor. Early detection will result in better survival. So, there should be a high index of suspicion when a vulvar lesion is seen, even if the patient falls below the typical age range and does not carry any well-known risk factors such as HPV infection and immunodeficiency.

## 1. Introduction

Invasive squamous cell carcinoma (ISCC) of the vulva is a rare disease and reflects 3–5% of all cancers of the female genital tract. Characteristically, it occurs in older women and is rarely seen in women younger than 35 years. However, in recent years, the incidence has been increasing in women younger than 50 years of age [1]. Vulvar ISCC can be classified into two groups according to pathogenic mechanism: basaloid (warty) carcinoma and keratinizing carcinoma. Keratinizing types are usually unifocal. They occur in older patients and originate from lichen sclerosus or squamous hyperplasia. Human papilloma virus (HPV) is not associated with keratinizing types of vulvar ISCC. Basaloid (warty) types are usually multifocal and associated with HPV, vulvar epithelial neoplasia (VIN), and smoking [2].

Some studies have shown that hypertension, obesity, diabetes mellitus, and nulliparity are risk factors for vulvar cancer [2]. Immune suppression has also been suggested as an important predisposing factor for the development of vulvar cancer in women of a younger age [3].

A persistent, itchy, ulcerous lesion may suggest vulvar cancer, but the only way to be certain that cancer is present is to do a vulvar biopsy.

Herein, we present a very rare case of HPV-negative ISSC of the vulva in an adolescent without any risk factors.

## 2. Case Presentation

**Figure 1:**
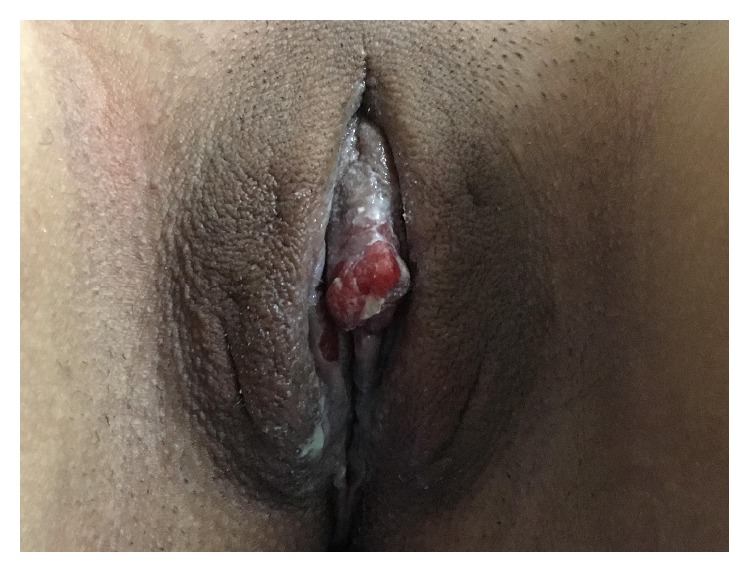
A localized and well-demarcated lesion on the labia minora.

**Figure 2:**
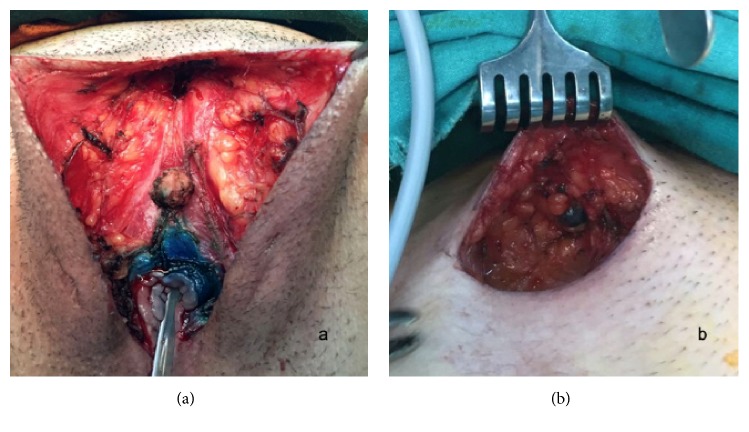
Vulvar excision and groin incision. (a) Vulvar defect after radical local excision. (b) Sentinel lymph node in the right inguinal region.

**Figure 3:**
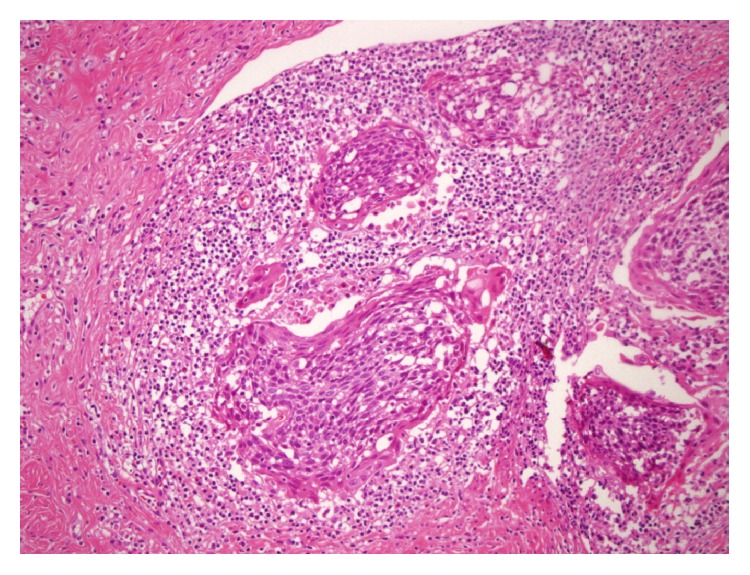
HE ×100. Nests of neoplastic squamous epithelium with keratinization.

**Figure 4:**
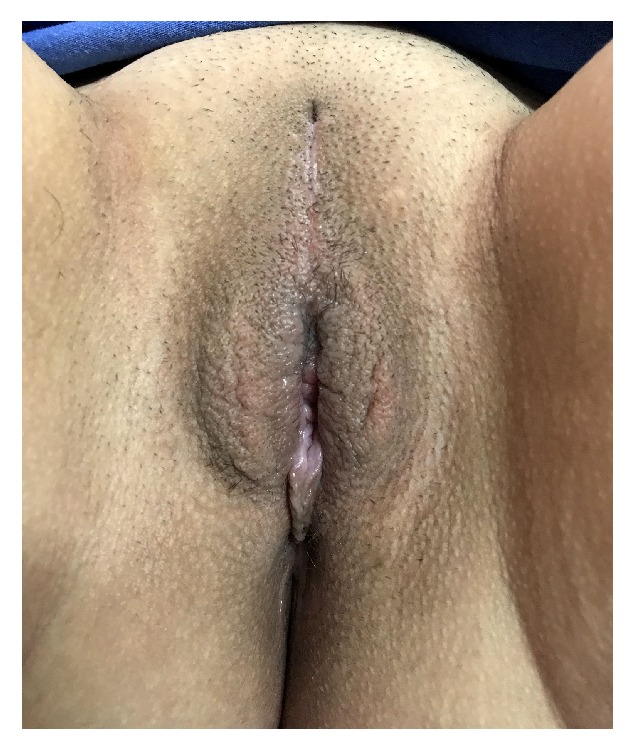
Surgical site recovery 12 months after surgery.

An 18-year-old patient presented with a pruritic, painful lesion on the vulva. There was no history of systemic disease, operation, or tobacco-alcohol use. She reported that she had been sexually active for one year. Physical examination revealed a hyperpigmented and erosive lesion, approximately 2 cm in diameter, centrally localized in the vulva (Figure 1). Inguinal lymph nodes were not palpable. Excisional biopsy was performed, and the result was reported as grade II keratinizing type ISCC of the vulva. There was no lymphovascular space invasion. Positron emission tomography revealed no nodal or distant metastasis. Pap and HPV tests returned negative results. Colposcopy was performed and normal colposcopic findings were observed. Vulvar radical local excision and bilateral inguinal sentinel lymph node biopsies (using methylene blue dye) were performed (Figure 2). The pathology report was similar to the excisional biopsy report with a clear surgical margin (Figure 3). Ultrastaging immunohistochemical evaluation was performed in all sentinel lymph nodes (2 in right inguinal region, 1 in left inguinal region) and no metastasis was seen. Immunohistochemical study of the surgical specimen was performed using anti-HPV mouse monoclonal antibody (Clone K1H8, 1:50; Dako, Carpinteria, CA) and the test was negative for HPV. Patient did not receive any adjuvant treatment. Follow-up after 12 months of the surgery showed no evidence of disease (Figure 4).

Written informed consent was obtained from the patient for publication of the case.

## 3. Discussion

The ISCC of the vulva is very rarely seen in patients under 35. The typical patients are 65–90 years old [4]. Recent literature lacks large data on ISCC of the vulva in young women. The youngest patient in a retrospective review of 18 patients younger than 45 years of age with vulvar cancer was 29 years old [5]. Another case series of 21 patients younger than 40 years of age included one patient aged 17 years with ISCC of the vulva. Although we are a referral center and performed more than 100 vulvar cancer surgeries in last 10 years, this patient was the youngest vulvar cancer case seen in our clinic so far. Depressed immunity has been suggested as the major predisposing factor in the development of vulvar ISCC in young women [3]. In the present case, there was no evidence of any immunosuppressive condition.

HPV-positive tumors are expected to occur more often in younger patients than HPV-negative tumors [6]. On the contrary, a previous study showed no statistically significant age difference in HPV or non-HPV cases [7]. In our patient, immunohistochemical study for HPV in vulvar excision specimen turned negative. At the same time, the vulvar tumor was unexpectedly keratinizing ISCC. One possible explanation is that most of vulvar ISCCs show keratinization and subjective nature of classifying the invasive component [3].

A SEER data study comparing vulvar cancer in younger versus older women found that, in younger women, disease is diagnosed at early stage and more likely to have surgical treatment without any adjuvant treatment [8]. In our case, the invasion of the tumor was greater than 1 mm; we performed radical local excision. Because the tumor had central localization, bilateral inguinal sentinel lymphadenectomy was performed. Sexual outcome of vulvar cancer treatment in young patients should be taken into consideration. The clitoris was protected during the surgery and vagina was not excised at all. As the surgical margins were negative and there was not sentinel lymph node metastasis on both sides, patient did not receive adjuvant therapy. The patient was satisfied with the final appearance of the vulva nine months after surgery.

Patients younger than 45 years with vulvar cancer tend to have early stage disease and a better prognosis [2]. Our patient had stage 1b disease. The 5-year survival rate of 90% was reported for stage I and II ISCC of the vulva [9].

In conclusion, there should be a high index of suspicion when a vulvar lesion is seen, even if the patient falls below the typical age range and does not carry any well-known risk factors such as HPV infection and immunodeficiency. A growing number of reports and studies on the ISCC of the vulva in young women are required.
